# A multi-ingredient, pre-workout supplement is apparently safe in healthy males and females

**DOI:** 10.3402/fnr.v59.27470

**Published:** 2015-06-16

**Authors:** Jordan M. Joy, Ryan P. Lowery, Paul H. Falcone, Roxanne M. Vogel, Matt M. Mosman, Chih-Yin Tai, Laura R. Carson, Dylan Kimber, David Choate, Michael P. Kim, Jacob M. Wilson, Jordan R. Moon

**Affiliations:** 1 MusclePharm Sports Science Institute, MusclePharm Corp., Denver, CO, USA; 2 Health Science and Human Performance Department, University of Tampa, Tampa, FL, USA; 3 Human Performance and Sport, Metropolitan State University of Denver, Denver, CO, USA; 4 Department of Sports Exercise Science, United States Sports Academy, Daphne, AL, USA

**Keywords:** caffeine, hematology, toxicity, nitrates, sports supplement

## Abstract

**Background:**

Pre-workout supplements (PWS) have become increasingly popular with recreational and competitive athletes. While many ingredients used in PWS have had their safety assessed, the interactions when combined are less understood.

**Objective:**

The purpose of this study was to examine the safety of 1 and 2 servings of a PWS.

**Design:**

Forty-four males and females (24.4±4.6 years; 174.7±9.3 cm; 78.9±18.6 kg) from two laboratories participated in this study. Subjects were randomly assigned to consume either one serving (G1; *n*=14) or two servings (G2; *n*=18) of PWS or serve as an unsupplemented control (CRL; *n*=12). Blood draws for safety panels were conducted by a trained phlebotomist before and after the supplementation period.

**Results:**

Pooled data from both laboratories revealed significant group×time interactions (*p*<0.05) for mean corpuscular hemoglobin (MCH; CRL: 30.9±0.8–31.0±0.9 pg; G1: 30.7±1.1–30.2±0.7 pg; G2: 30.9±1.2–30.9±1.1 pg), MCH concentration (CRL: 34.0±0.9–34.4±0.7 g/dL; G1: 34.1±0.9–33.8±0.6 g/dL; G2: 34.0±1.0–33.8±0.8 g/dL), platelets (CRL: 261.9±45.7–255.2±41.2×10^3^/µL; G1: 223.8±47.7–238.7±49.6×10^3^/µL; G2: 239.1±28.3–230.8±34.5×10^3^/µL), serum glucose (CRL: 84.1±5.2–83.3±5.8 mg/dL; G1: 86.5±7.9–89.7±5.6 mg/dL; G2: 87.4±7.2–89.9±6.6 mg/dL), sodium (CRL: 137.0±2.7–136.4±2.4 mmol/L; 139.6±1.4–140.0±2.2 mmol/L; G2: 139.0±2.2–138.7±1.7 mmol/L), albumin (CRL: 4.4±0.15–4.4±0.22 g/dL; G1: 4.5±0.19–4.5±0.13 g/dL; G2: 4.6±0.28–4.3±0.13 g/dL), and albumin:globulin (CRL: 1.8±0.30–1.8±0.28; G1: 1.9±0.30–2.0±0.31; G2: 1.8±0.34–1.8±0.34). Each of these variables remained within the clinical reference ranges.

**Conclusions:**

The PWS appears to be safe for heart, liver, and kidney function in both one-serving and two-serving doses when consumed daily for 28 days. Despite the changes observed for select variables, no variable reached clinical significance.

Among both recreational and professional athletes, the prevalence of supplementation has grown. It is estimated that 48–53% of Americans use supplements on a regular basis, often citing overall health and wellness as reasons for supplementation (1). Moreover, the practice of ‘nutrient timing’ is becoming increasingly popular for the purposes of improved cognition, performance, and recovery (2). One of the more popular timing windows for supplementation includes the period immediately prior to the exercise bout.

Pre-workout supplements (PWS) generally contain multiple ingredients that claim to provide ergogenic benefits. Many athletes believe that supplementation prior to training will result in improved strength, focus, and enhanced training adaptations. In a study conducted by Spradley et al., it was discovered that consuming a PWS 20 min before exercise improved reaction time, improved muscular endurance, and delayed fatigue (3). Another study investigated the effects of 8 weeks of supplementation with a PWS containing caffeine, creatine, and branched chain amino acids (BCAAs) and found that those consuming the supplement had greater increases in lean mass, quadriceps muscle thickness, and bench press strength compared with placebo (4). Kraemer et al. observed improvements in vertical jump power, fatigue resistance, anabolic hormone profiles, and ratings of perceived exertion following 7 days supplementation involving caffeine, creatine, and amino acids 30 min prior to their self-selected exercise as well as 30 min prior to each testing day (5). Similarly, Kedia and colleagues reported improvements in perceived energy and concentration with PWS supplementation containing creatine, betaine, and caffeine, but they did not observe significant interactions for body composition or performance. Moreover, the PWS did not produce changes in hematology, and the researchers concluded the supplement as safe (6). Finally, a 28-day assessment of a PWS containing caffeine, creatine, and beta-alanine observed increases in strength along with no unusual changes in blood chemistry, indicating its safety (7).

Dietary nitrates have recently grown in popularity, particularly in the form of beetroot juice. Nitrates have been demonstrated to improve performance (8, 9) by increasing muscle contractile efficiency via decreased oxygen and overall energy cost of exercise (10, 11). Bailey et al. discovered that this may likely be due to a decreased ATP cost of muscle contraction following nitrate supplementation (10). More recently, nitrates have been supplemented in the form of nitrate-bound ingredients, such as the BCAAs and creatine (12). The BCAAs, particularly leucine, have been known to increase muscle protein synthesis (13), and as a product, they enhance recovery and reduce muscle soreness (14). Thus, nitrate-bound BCAAs may enhance both exercise performance and exercise recovery. Despite reported health benefits of nitrate supplementation (15, 16), concerns have been raised over nitrosamines (17, 18). Nitrate-bound creatine has been reported as safe (12), yet the safety of nitrate-bound amino acids is yet to be examined.

Although these and other common ingredients found in PWS, such as amino acids and beta-alanine, have been independently studied, the combination of these ingredients and their effects on blood chemistry, blood pressure (BP), and heart rate (HR) are not well understood. Various combinations of these ingredients exist in the marketplace, and it is important to determine the safety of each of these combinations particularly novel ingredients, such as those bound to nitrate. Moreover, as consumers become caffeine tolerant after chronic use and/or believe in the ‘more is better’ approach, individuals may choose to increase their dosage. However, little is known about the safety of consuming more than one serving of a PWS relative to a control and one serving. Therefore, the purpose of this investigation is to determine the safety of ingesting 1 and 2 servings of a PWS containing caffeine, creatine, nitrate-bound amino acids, beta-alanine, and vitamins for 28 days in recreationally trained males and females. We hypothesize that the PWS will produce no changes in hematological safety parameters indicative of heart, liver, and kidney health or vital signs.

## Methods

### Experimental design

**Fig. 1:**
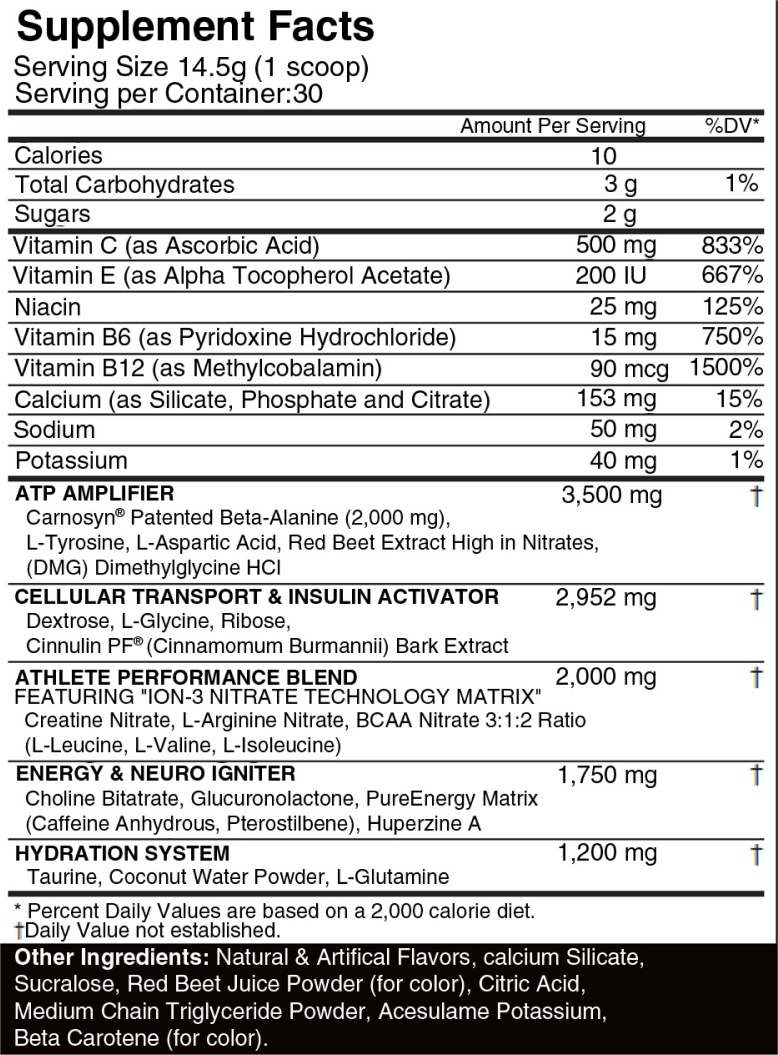
Supplement facts. Each serving contains beta-alanine, creatine, amino acids, caffeine, and approximately 700–800 mg of dietary nitrate.

In a randomized design, a total of 44 subjects were recruited for this study across two laboratories. Subjects were randomly divided into control (CRL), one-serving (G1) group, or two-serving (G2) groups. Wherein, the CRL group did not supplement and G1 and G2 consumed one serving and two servings of the PWS (Assault™, MusclePharm Inc., Denver, CO), respectively, every day for 28 days in an unblinded manner. The contents of the PWS can be found in Fig. 1. Supplement containers were weighed prior to and following the supplementation period and supplement consumption logs were completed by each participant to ensure compliance. G1 was 96% and G2 was 100% compliant according to supplement weight, equating to 98% for all treatment conditions. Blood draws and vital signs were conducted prior to and at the conclusion of the supplementation period. Throughout the trial period, subjects completed 3-day food logs each week. Subjects were instructed to maintain their current diets, and diets remained unchanged throughout the duration of the trial. MusclePharm Sports Science Institute (Lab 1) received approval from its institutional review board (IRB), and the University of Tampa Human Performance and Nutrition Laboratory (Lab 2) received approval from the University of Tampa IRB. Each subject was provided written informed consent prior to participation in the study.

### Participants

Twenty-seven subjects (26.2±5.0 years, 174.3±10.6 cm, 81.2±21.3 kg, CRL *n*=7 males, 5 females, G1 *n*=2 males, 4 females, G2 *n*=6 males, 3 females) were recruited by Lab 1, while Lab 2 recruited the remaining 17 (21.6±1.3 years, 175.3±7.0 cm, 75.5±13.2 kg, G1 *n*=5 males, 4 females, G2 *n*=5 males, 3 females). Two laboratories were used to both increase sample size as well as demonstrate differences between laboratories using similar populations in different areas of the country. Additionally, the inclusion of an independent third-party strengthens the credibility of the study for those who may perceive a conflict of interest. Subjects were required to be at least recreationally active (≥3 days/week of moderate to vigorous intensity exercise), free of any disease or disorder which may produce confounding results, non-smokers, and to have abstained from supplements other than a multi-vitamin, protein powder, and naturally occurring caffeine sources such as coffee and tea, as assessed by pre-participation health history, exercise, and supplementation questionnaires. Subjects reported an average daily caffeine intake of 433 mg for G1 and 271 mg for G2 prior to study enrollment.

### Measurements

All measurements were taken prior to and at the conclusion of the 28-day supplementation period. Following a 10-h fast, all subjects submitted a blood sample for analysis in the morning to prevent diurnal variations. All blood draws were performed via venipuncture by a trained phlebotomist. Samples were analyzed for complete metabolic panels and complete blood counts by an external laboratory (Laboratory Corporation of America, Denver, CO; ANY LAB TEST NOW, Tampa, FL). All collected samples were analyzed for the following markers: white blood cell count (WBC), red blood cell count (RBC), hemoglobin, hematocrit, mean corpuscular volume (MCV), mean corpuscular hemoglobin (MCH), mean corpuscular hemoglobin concentration (MCHC), red blood cell distribution width (RDW), platelets (percent and absolute), neutrophils (percent and absolute), lymphocytes (percent and absolute), monocytes (percent and absolute), eosinophils (percent and absolute), basophils (percent and absolute), serum glucose, blood urea nitrogen (BUN), creatinine, estimated glomerular filtration rate (eGFR), BUN: creatinine, sodium, potassium, chloride, carbon dioxide, calcium, protein, albumin, globulin, albumin:globulin, bilirubin, alkaline phosphatase, aspartate aminotransferase (AST), and alanine aminotransferase (ALT). BP and heart rate (HR) were recorded at the brachial artery using an automatic, digital sphygmomanometer (Omron Corporation, Kyoto, Japan). Vital signs were measured prior to the blood draw following 10 min stationary rest by the participants, and measurements were conducted in the seated position while the arm rested on a table such that the brachial artery was level with the heart. Intra-test Coefficient of Variation (CV) for Lab 2 blood measurements were all under 3%. Inter-test reliability results from 12 men and women measures up to 1 week apart from Lab 1 resulted in no significant differences from day-to-day (*p*>0.05) and an average inter-test %CV of 6.9%.

### Statistical analyses

Pooled data were analyzed using a 3×2 repeated measures ANCOVA model for all group, time, and group by time interactions with the pre-value as the covariate. A Bonferroni post-hoc analysis was used to locate differences. Shapiro–Wilk tests were used to determine normality of the data. The Minimal Difference (MD) needed to be considered real was determined using the method previously described by Weir (19). Data are presented as mean±standard deviation. All data were analyzed using Statistica software (Statsoft, Tulsa, OK, 2011).

**Table 1:** Diet and Exercise Data. Data are presented as mean±standard deviation

Variable	Treatment	Week 1	Week 2	Week 3	Week 4
Calories (kcal/day)	CRL	1816.1±627.8	1879.6±718.9	1668.2±773.0	1848.2±581.7
	G1	2112.8±625.0	2118.8±571.7	2167.8±548.8	2247.2±512.1
	G2	2345.1±510.7	2297.6±508.8	2397±695.7	2537.1±618.0
Carbohydrates (g/day)	CRL	171.1±81.5	172.4±55.1	152.9±51.7	163.6±71.6
	G1	243.8±95.5	246.6±95.2	247.2±87.8	264.9±70.8
	G2	258.5±91.4	254.5±84.9	271.3±88.3	282.7±64.1
Fats (g/day)	CRL	66.6±26.9	67.8±32.7	70.8±33.1	76.0±25.6
	G1	73.5±21.7	72.8±22.8	74.2±22.9	77.1±20.9
	G2	78.5±23.2	79.3±24.6	80.5±37.1	87.0±35.9
Protein (g/day)	CRL	115.6±32.2	110.7±59.7	119.9±62.7	122.9±66.2
	G1	118.6±21.1	119.4±25.5	129.0±29.2	128.1±18.4
	G2	139.2±36.2	140.7±36.4	147.9±47.1	1503±51.6
Exercise (days/week)	CRL	5.9±1.2	5.6±0.7	4.9±1.8	4.8±1.4
	G1	4.8±1.6	4.3±1.2	3.8±1.2	5.2±1.5
	G2	5.8±1.5	6.0±0.9	5.8±1.2	5.7±1.0
Exercise (minutes/week)	CRL	373.2±149.3	382.3±138.8	315.6±163.0	268.1±101.9
	G1	360.8±135.6	240.8±83.2	254.2±90.4	340.0±154.6
	G2	541.7±222.2	540.8±233.8	570.8±232.0	470.8±174.9

## Results

### Diet and exercise

Diets did not change throughout the trial between groups. No significant group×time interactions were observed for average daily calories between weeks (*p*=0.47), carbohydrates (*p*=0.25), fat (*p*=1.00), or protein (*p*=0.98). Similarly, no changes were observed for activity levels throughout the trial. No significant group×time interactions were observed for days exercised per week (*p*=0.52) or minutes exercised per week (*p*=0.053). Data are presented in Table 1.

### Pooled

Significant group×time interactions were present for MCH, in which G1 decreased over time and relative to CRL and G2. Significant group×time interactions were observed for MCHC. Wherein, G1 and G2 decreased while CRL increased. Significant group×time interactions were present for platelets, in which G1 increased from pre to post and relative to CRL, and G2 decreased significantly compared to CRL. Platelets were normally distributed at baseline (*p*=0.36) but positively skewed at post (*p*<0.05). Significant group×time effects occurred for serum glucose, in which G1 and G2 increased significantly versus CRL. Significant group by time effects were observed for sodium. Wherein, G1 increased and G2 decreased relative to CRL. Significant group×time interactions were present for albumin, which decreased significantly in G2 from pre to post and relative to CRL. Significant group×time interactions existed for albumin:globulin, in which G1 increased versus CRL and G2. Albumin:globulin was positively skewed at baseline (*p*<0.05), but it was normally distributed at post (*p*=0.12). MCH, MCHC, serum glucose, and sodium were normally distributed at both time points (*p*>0.05). Albumin was negatively skewed at both time points (*p*<0.05). Means and standard deviations can be found in Supplementary File 1.

### Inter-laboratory comparisons: one serving

Significant lab×time effects were observed for HR, in which CRL and Lab 2 increased over time and relative to G1, and Lab 2 increased to a greater extent than CRL. The distribution for HR shifted from normal at baseline (*p*=0.40) to a negative skew at post (*p*<0.05). Significant lab×time interactions were present for WBC. Wherein, Lab 2 increased relative to both CRL and Lab 1. WBC was normally distributed at baseline (*p*=0.06) but negatively skewed at post (*p*<0.05). A significant lab×time effect existed for MCH, in which Lab 2 decreased relative to CRL. Significant lab×time interactions were observed for MCHC. Wherein, Lab 2 decreased relative to CRL and over time. Significant lab×time effects were observed for platelets, which increased for Lab 2 relative to CRL and Lab 1, and increased for Lab 1 relative to CRL. Significant lab×time interactions were present for absolute lymphocytes. Wherein, Lab 2 increased significantly over time and relative to CRL. Significant lab×time interactions existed for absolute monocytes, in which Lab 2 increased over time and relative to CRL and Lab 1. Absolute monocytes were positively skewed at baseline (*p*<0.05) and negatively skewed at post (*p*<0.05). Significant lab×time interactions were observed for carbon dioxide. Wherein, CRL decreased over time and relative to Lab 1 and Lab 2, and Lab 2 decreased to a greater extent than Lab 1. Significant lab×time interactions were present for calcium, in which CRL decreased relative to Lab 1, and Lab 2 increased relative to CRL and Lab 1. Calcium was normally distributed at baseline (*p*=0.29), but it became negatively skewed at post (*p*<0.05). MCH, MCHC, platelets, absolute lymphocytes, and carbon dioxide were normally distributed at each time point (*p*>0.05). Means and standard deviations can be found in Supplementary File 2.

### Inter-laboratory comparisons: two servings

Significant lab×time effects were observed for HR, in which CRL and Lab 2 increased over time, and Lab 2 increased relative to CRL and Lab 1. HR was normally distributed at baseline (*p*=0.47) yet negatively skewed at post (*p*<0.05). Significant lab×time effects were present for MCHC. Wherein, CRL increased relative to Lab 1 and Lab 2. Significant lab×time interactions existed for serum glucose. Wherein, Lab 2 increased relative to CRL and Lab 1. Significant lab×time effects were observed for sodium, in which Lab 2 decreased to a greater extent than CRL and Lab 1. MCHC, serum glucose, and sodium were normally distributed at each time point (*p*>0.05). Means and standard deviations can be found in Supplementary File 3.

## Discussion

The results of the present study confirm the hypothesis that 28 days PWS supplementation appears not to cause abnormal changes in hematological safety markers. When analyzing the pooled data from both laboratories, significant interactions were observed only for MCH, MCHC, platelets, serum glucose, sodium, albumin, and albumin:globulin. However, each of these markers remained within the clinical reference range. Moreover, platelets and albumin:globulin had differing distributions between time points, thereby increasing the probability for type 1 statistical error (20). Additionally, MCH, MCHC, platelets, sodium, and albumin:globulin demonstrate unusual trends between groups. For each of these, G1 had either changes of higher magnitude than G2 or changes in a different direction, and MCHC had a greater magnitude of change in CRL compared to both G1 and G2. Collectively, these results suggest a natural variation for these markers. Another contributing factor to variation could be attributed to the differences in environment (21). This would also help to explain the differences observed between laboratories for HR, WBC, MCH, MCHC, platelets, absolute lymphocytes, absolute monocytes, carbon dioxide, and calcium. Similar to the pooled analysis, some of these may be due to type 1 statistical error, as HR in the one- and two-serving inter-laboratory analyses and WBC, absolute monocytes, and calcium in the one-serving inter-laboratory analysis each had differences in their distribution between pre- and post-measurements. Furthermore, MCH, MCHC, serum glucose, and platelets reaching significance in the pooled analysis may be partially caused by the variation between laboratories, as the CRL group was composed of only those from Lab 1.

Analysis of clinical significance at the individual level was conducted using the MD statistic that calculates the (biological and reliability) error needed to be exceeded in order for an individual change score to be considered real as described by Weir (19). If a subject exceeded the MD, the change was considered a true change. Variables that were significantly different at the group level were evaluated at the individual level and subjects with changes that exceeded MD were evaluated to determine clinical significance. Clinical significance at the individual level was reached when a score that exceeded the MD crossed the upper or lower limits for each variable. For the pooled analysis, one participant from Lab 2 in G2 increased, leaving the range for serum glucose. However, one individual is not enough evidence to indicate a change due to supplementation. More evidence for variability is found when examining sodium, for which two participants from CRL experienced clinical change; one decreased to leave the range and one increased to enter the range. No individuals crossed the upper or lower limit for MCH, MCHC, platelets, albumin, or albumin:globulin.

The present findings agree with previous literature. Shelmadine et al. (22) determined that a PWS containing caffeine, creatine, beta-alanine, and amino-acids was safe for consumption based upon clinical blood chemistry markers. Similar in design to the present study, these researchers supplemented study participants daily with a PWS or placebo for 28 days and collected blood samples prior to and after the supplementation period. In contrast to the present findings, these researchers observed no changes for any marker. This is in agreement with another study conducted by Kendall and colleagues, who found that 28 days of supplementation with a PWS is safe (7). In a 9-week study of a multi-ingredient product consumed pre- and intra-workout, no changes were observed for clinical safety markers, BP, or HR (23).

While significant changes in clinical markers were not expected, it was assumed that if changes were to occur that they would be most robust in G2. However, under this assumption, the pooled analysis further suggests changes due to the natural variability of hematological markers. G1 experienced significant changes in MHC that G2 did not; for MCHC, CRL decreased more than G1 or G2 increased; and G1 and G2 had divergent changes for platelets and sodium with G1 increasing and G2 decreasing. Only serum glucose and albumin may suggest a stronger effect for G2.

The present PWS is composed of several nitrate-containing ingredients, which may worry some athletes or practitioners. Several decades ago, nitrosamines were proposed as possible carcinogens (18). While more recent evidence has indicated the contrary (24), the stigma associated with nitrates persists. In a review of nitrate supplementation, Hoon and colleagues (25) reported no major health consequences of nitrate supplementation, and only one minor adverse event, the discoloration of urine, which is attributed to those studies which supplemented with beetroot juice. Moreover, dietary nitrate has been reported to reduce BP on several occasions (16, 26–28). However in the present study, the PWS did not seem to augment BP; rather, it appears that HR may potentially increase in some subjects as demonstrated by the Lab 2 data subset. The present study also examined a relatively high dose of nitrate (approximately 700–800 mg or 11.3–12.9 mM per serving) compared to previous studies using 5.1–11.2 mM (10, 27, 29) versus 22.6–25.8 mM used in G2.

### Limitations

One primary concern of exogenous nitrates is an increased risk for cancer. While we were able to measure several common clinical safety markers, we were unable to determine methemoglobin and nitrosamine formation. However, it does not appear that the supplement influences heart, liver, or kidney function. The duration of this study was 28 days. Therefore, very long term effects of PWS supplementation (e.g. 1 year) cannot be determined.

## Conclusions

Novel to this study is the examination of nitrate-bound amino acids and creatine. Our laboratories have recently reported on the safety of creatine nitrate (12), finding it to be safe in doses of both 1 and 2 g for 28 days. However, the safety of nitrate-bound amino acids had not been previously examined. Moreover, previous literature has not reported on the safety of nitrate in doses approximately 400% that of an efficacious dose for performance enhancement. It appears from the present study that dietary nitrates from nitrate-bound amino acids are safe for heart, liver, and kidney function after 28 days consumption in healthy males and females in conjunction with the other ingredients found in the PWS. This was the first study to assess the safety of nitrate-bound BCAAs used in combination with other ergogenic ingredients. From the present results, we can conclude that the PWS is safe for up to 28 days consumption in healthy, recreationally active males and females. Additionally, two servings of a PWS appear to be just as safe as one serving. Moreover, dietary nitrate, in doses of up to approximately 1,500 mg, seems to be safe for human consumption for a period of up to 28 days in this population. Despite significant interactions for MCH, MCHC, platelets, serum glucose, albumin, and albumin:globulin, the magnitude of change was small, and all measured variables remained within the normal range across all groups. Future research may be interested in examining the PWS for a longer trial period to confirm its safety, to determine methemoglobin and nitrosamine formation, and to determine the ergogenic capacity of the PWS.

## Supplementary Material

A multi-ingredient, pre-workout supplement is apparently safe in healthy males and females

A multi-ingredient, pre-workout supplement is apparently safe in healthy males and females

A multi-ingredient, pre-workout supplement is apparently safe in healthy males and females
